# *IOTA*: integration optimization, triage and analysis tool for the processing of XFEL diffraction images^1^

**DOI:** 10.1107/S1600576716006683

**Published:** 2016-05-11

**Authors:** Artem Y. Lyubimov, Monarin Uervirojnangkoorn, Oliver B. Zeldin, Aaron S. Brewster, Thomas D. Murray, Nicholas K. Sauter, James M. Berger, William I. Weis, Axel T. Brunger

**Affiliations:** a Department of Molecular and Cellular Physiology, Stanford University, 318 Campus Drive, Stanford, CA 94305, USA; b Department of Neurology and Neurological Science, Stanford University, 318 Campus Drive, Stanford, CA 94305, USA; c Department of Structural Biology, Stanford University, 318 Campus Drive, Stanford, CA 94305, USA; d Department of Photon Science, Stanford University, 318 Campus Drive, Stanford, CA 94305, USA; e Howard Hughes Medical Institute, Stanford University, Stanford, CA 94305, USA; f Molecular Biophysics and Integrated Bioimaging Division, Lawrence Berkeley National Laboratory, Berkeley, CA 94720, USA; g Biophysics Graduate Group, University of California, Berkeley, CA 94720, USA; h Department of Biophysics and Biophysical Chemistry, Johns Hopkins University School of Medicine, Baltimore, MD 21205, USA

**Keywords:** diffraction data processing, X-ray free-electron lasers, XFELs, serial femtosecond crystallography, indexing and integration, computer programs

## Abstract

Serial femtosecond crystallography (SFX) uses an X-ray free-electron laser to extract diffraction data from crystals not amenable to conventional X-ray light sources owing to their small size or radiation sensitivity. However, a limitation of SFX is the high variability of the diffraction images that are obtained. As a result, it is often difficult to determine optimal indexing and integration parameters for the individual diffraction images. Presented here is a software package, called *IOTA*, which uses a grid-search technique to determine optimal spot-finding parameters that can in turn affect the success of indexing and the quality of integration on an image-by-image basis. Integration results can be filtered using *a priori* information about the Bravais lattice and unit-cell dimensions and analyzed for unit-cell isomorphism, facilitating an improvement in subsequent data-processing steps.

## Abstract 2

An integration optimization, triage and analysis tool (*IOTA*) is presented, which uses a grid-search approach to maximize the success of indexing and integrating serial X-ray free-electron laser diffraction images. *IOTA* also includes several useful tools for on-site diffraction data processing.

## 1. Introduction   

X-ray free-electron lasers (XFELs) (Emma *et al.*, 2010 ▸) enable serial femtosecond crystallography (SFX) using powerful ultra-fast (5–50 fs) highly focused X-ray laser pulses with a beam diameter as small as 100 nm (Liang *et al.*, 2015 ▸). These properties facilitate diffraction data collection from crystals that are too small (<5 µm) and/or too radiation sensitive for data collection at standard synchrotron light sources (Schlichting, 2015 ▸). The ultra-short duration of the XFEL pulse allows collection of a diffraction pattern before significant radiation damage occurs (Neutze *et al.*, 2000 ▸; Solem, 1986 ▸). Since the exposed volume is generally vaporized by the XFEL pulse, this process is referred to as ‘diffraction before destruction’ (Chapman *et al.*, 2006 ▸); as a consequence, each diffraction image must be collected from a different crystal or crystal volume using a liquid-jet sample-delivery system (Chapman *et al.*, 2011 ▸; Sierra *et al.*, 2012 ▸; Weierstall, 2014 ▸) or a fixed-target technique (Hunter *et al.*, 2014 ▸; Cohen *et al.*, 2014 ▸; Zhou *et al.*, 2015 ▸). The variation in crystal size, quality and mosaic structure and in their position and orientation with respect to the XFEL beam, and the shot-to-shot variation in energy spectrum and intensity produced by the self-amplified spontaneous emission (SASE) process, result in high variability in the recorded diffraction images (Hattne *et al.*, 2014 ▸; Kern *et al.*, 2012 ▸, 2013 ▸; Schlichting, 2015 ▸; Sauter, 2015 ▸). Furthermore, the difficulties inherent in processing serial diffraction data cause a large percentage of diffraction images to be discarded by the data-processing software, which often affects the quality – or overall usefulness – of the merged diffraction data set.

As has been recently shown, the number of raw diffraction images necessary for a complete data set can be greatly reduced by the use of post-refinement of integrated intensities (Gin *et al.*, 2015 ▸; Kabsch, 2014 ▸; Kroon-Batenburg *et al.*, 2015 ▸; Uervirojnangkoorn *et al.*, 2015 ▸; White, 2014 ▸). This technique, however, performs best when a reasonably complete set of integrated diffraction images is available, which in turn requires an optimal quality of integration for each diffraction image. Even when a diffraction image is successfully integrated, the Bravais lattice and/or unit-cell parameters may be inaccurately determined, especially in cases of poor diffraction. Such a failure to integrate the data with the optimal lattice model will adversely affect the quality of the merged and post-refined data set (Sauter, 2015 ▸) and, consequently, the quality of the resulting electron-density maps. Thus, detecting crystal non-isomorphism, lack of diffraction or mis-indexed frames prior to merging and post-refinement would substantially improve the quality of the diffraction data and refined atomic models obtained from SFX experiments.

The variability in the diffraction images leads to a number of challenges in SFX data processing. Specifically, the indexing step, whereby the Bravais lattice, unit-cell parameters and orientation of the crystal are determined, is challenging for these zero-rotation (or ‘still’) diffraction images. Currently in *cctbx.xfel*, one of the program suites used to process XFEL images (Hattne *et al.*, 2014 ▸), indexing is carried out individually for each diffraction image. Indexing is heavily dependent on the success of the preceding ‘spot-finding’ step, carried out in *DISTL* (Zhang *et al.*, 2006 ▸), in which candidate reflections at Bragg positions are located in the raw diffraction image. Unreliable spot finding exacerbates the inherent difficulty of unambiguously determining crystal orientation from each still diffraction image (Sauter *et al.*, 2014 ▸) which, combined with errors in the positional accuracy of the detector (Hattne *et al.*, 2014 ▸; Sauter, 2015 ▸), leads to large uncertainties in the lattice models. In the present state-of-the-art software, these uncertainties are not easy to remove, so the fidelity of the starting lattice model obtained from the initial set of Bragg spots is of utmost importance to the quality of the final integrated intensities (Sauter, 2015 ▸). Moreover, spot-finding results are also used to estimate the limiting diffraction resolution and to generate reflection integration masks, which are used to separate signal from noise (Hattne *et al.*, 2014 ▸; Sauter, 2015 ▸).

**Figure 1:**
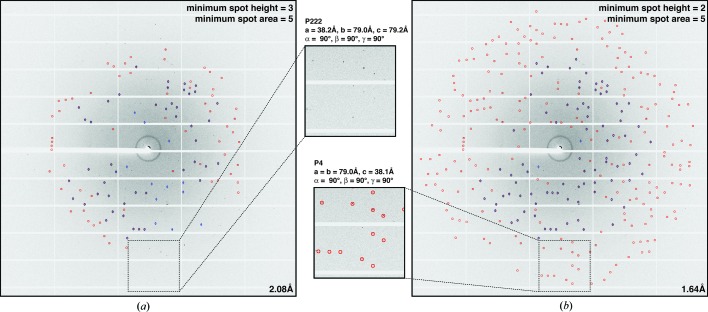
The importance of spot-finding parameters for indexing and integration. Integration results are shown for a representative diffraction image (using synchrotron radiation and a Dectris PILATUS 6M detector) from hen egg-white lysozyme (HEWL) with different spot-finding parameters. Spot-finding results are marked as blue diamonds, while integration predictions are marked as red circles. No target unit cell was used, in order to emphasize the effect of spot finding on indexing and integration. (*a*) Indexing and integration using a minimum spot height of 3σ and a minimum spot area of 5 pixels (inset: clearly visible diffraction reflections that were not integrated). (*b*) Indexing and integration using a minimum spot height of 2σ and a minimum spot area of 5 pixels [inset: the same diffraction reflections as in part (*a*), but now clearly marked for integration]. Note how a small difference in the minimal spot-height parameter results in a small increase in the number of spots found (58 with spot height = 3, 85 with spot height = 2), which in this case leads to a slightly better lattice model and a dramatic difference in indexing and integration results.

Optimal spot finding depends on optimal spot-finding parameters. We found that, even with an excellent diffraction pattern from hen egg-white lysozyme (HEWL) crystals, just a slight change in spot-finding parameters could result in a profound difference in integration results (Fig. 1 ▸). In *cctbx.xfel*, the spot-finding parameters are user determined, typically by a trial-and-error process that uses visual inspection of a diffraction image, subjective evaluation of the results, and multiple iterations of the indexing and integration steps. Once the initial spot-finding parameters have been determined, they are then applied to all diffraction images in the data set. While this approach can provide reasonable results for the majority of data collected, it may result in sub-optimal processing of those diffraction images whose reflections, owing to crystal heterogeneity and XFEL pulse variations, are dimmer, brighter, larger, smaller or differently shaped than those used for the spot-finding parameter determination step.

Here, we present the program *IOTA* (integration optimization, triage and analysis), which uses raw diffraction images as input, converts them to a format readable by *cctbx.xfel* (Hattne *et al.*, 2014 ▸), performs indexing and integration using *cctbx.xfel* modules, and analyzes the integration results. *IOTA* uses a grid-search algorithm that quickly establishes a different set of optimal spot-finding parameters for each individual diffraction image in a fully automated manner. This approach substantially improves the integration efficiency and thus the overall quality of the resulting merged and post-refined data set (Sauter, 2015 ▸). Further improvements can also be made by including unit-cell and Bravais lattice filters, provided such information is available *a priori*, as well as by using a hierarchical unit-cell clustering method (Zeldin *et al.*, 2015 ▸) that can determine best-estimate average unit-cell dimensions from a group of crystals and resolve heterogeneities among the crystalline samples. Used together with the post-refinement program *PRIME* (Uervirojnangkoorn *et al.*, 2015 ▸), *IOTA* is part of a data-processing suite that has been used to obtain high-quality diffraction data sets even from a limited number of images, such as the XFEL-derived data set of the synaptotagmin-1/SNARE complex (Zhou *et al.*, 2015 ▸).

## 2. Program design   

### 2.1. Overall architecture   

**Figure 2:**
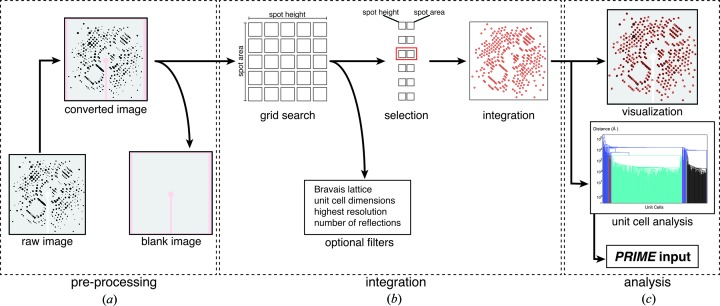
An overview of *IOTA*. (*a*) The diffraction image pre-processing module. Images are converted to a format compatible with *cctbx.xfel* (for example, the image needs to be modified such that the beam center coincides with the center of the image) and blank images are optionally discarded. (*b*) The integration module, with optional grid-search function to optimize spot-finding parameters, as well as optional filters that reduce the pool of integration results to those with a user-defined Bravais lattice, unit cell, resolution and number of reflections. (*c*) The analysis module, with hierarchical integration result visualization (top), unit-cell clustering (middle) and assembly of the *PRIME* input script (bottom).

*IOTA* is composed of three major modules (Fig. 2 ▸). The image preprocessing module imports, converts and triages raw diffraction images. The second module performs indexing and integration of diffraction images using *cctbx.xfel*. The third module performs diagnostics on the processed diffraction images. The modules are controlled by a command-line interface, which is assisted by two user-accessible parameter files: in one, the user supplies settings for *IOTA* itself, while the other contains spot-finding, indexing and integration parameters for *cctbx.xfel*. Initial (default) parameter files are generated automatically from the available diffraction images; the user can modify the parameter files if different settings are desired, or alter individual settings directly *via* command-line arguments.

### 2.2. Diffraction image triage and pre-processing   

XFEL-based diffraction data collection often yields images with no discernible diffraction, especially when liquid jet-based sample delivery or blind rastering techniques are employed, as laser pulses illuminate volumes with no crystal present. Often, as much as 90% or more of the diffraction data set is composed of such ‘blank’ images; attempts to index and integrate these images, while abortive, can greatly slow down data processing. Likewise, images with poor-quality diffraction that extends to very low resolution can impair an otherwise good merged diffraction data set. Thus, a ‘triage’ step, where images with poor or no diffraction are identified and discarded, is essential to ensure optimal and expeditious data processing. To that end, *cctbx.xfel* and *IOTA* utilize the program *DISTL* (Zhang *et al.*, 2006 ▸), which identifies potential Bragg spots. The user has the option of modifying some of the triage parameters (*e.g.* the minimum number of detected Bragg spots used to determine whether the image contains diffraction) or bypassing the triage step altogether.

Additionally, specific features of the detector geometry must be taken into account when processing diffraction images. Among the currently used detectors for SFX experiments at the SLAC Linac Coherent Light Source (LCLS; Stanford, California, USA) are the high-readout speed (120 Hz) Cornell–SLAC pixel-array detector (CSPAD) (Blaj *et al.*, 2015 ▸) and the charge-coupled device (CCD) detectors. The CSPAD detector is most frequently used for liquid jet-based experiments on the Coherent X-ray Imaging (CXI) endstation of LCLS. CCD detectors such as the Rayonix MX325-HE and MX170-HS are used for goniometer-based fixed-target experiments (Cohen *et al.*, 2014 ▸) on the X-ray Pump Probe (XPP) and Macromolecular Femtosecond Crystallography (MFX) endstations, and for other serial crystallography experiments at frame rates up to 10 Hz. There are several challenges inherent to each detector and experimental setup that need to be addressed prior to the main data-processing procedures.

The pinwheel layout of the CSPAD application-specific integrated circuits (ASICs) leads to sub-pixel shifts between individual panels, which can be accurately determined by a detector geometry refinement algorithm (Hattne *et al.*, 2014 ▸). The large beam stop necessary to protect the Rayonix MX325-HE detector from the direct XFEL pulse creates a shadow on the collected diffraction images, which interferes with indexing and integration and must be masked. Finally, the current version of the *cctbx.xfel* indexing module requires that the direct-beam coordinates coincide with the center of the image in order for indexing to be successful. When this is not the case (*i.e.* when the center of the detector is offset with respect to the incident beam), the image must be modified by adding or removing the requisite number of pixels along the edges.

Most of these pre-processing steps can be carried out using a number of tools available in the *cctbx.xfel* suite of software. To make the process more user friendly, *IOTA* automatically analyzes the incoming diffraction images and performs the necessary modification and triage steps (Fig. 2 ▸ *a*). Converted and modified images are written to a specially designated directory, and details of the pre-processing and triage steps are recorded in the log. *IOTA* also accepts as input diffraction images that have already been converted to *cctbx.xfel* format and/or modified using other software.

### 2.3. Diffraction image indexing and integration   

Indexing and integration of diffraction images are carried out using modules from the *cctbx.xfel* software suite (Hattne *et al.*, 2014 ▸). In *IOTA*, the user has the option of determining the optimal combination of spot-finding parameters for each individual diffraction image (Fig. 2 ▸ *b*) using an automated grid-search procedure, thus potentially improving the quality and quantity of individual integrated images, as well as the quality of the merged data set.

#### 2.3.1. Spot-finding parameter grid search   

**Figure 3:**
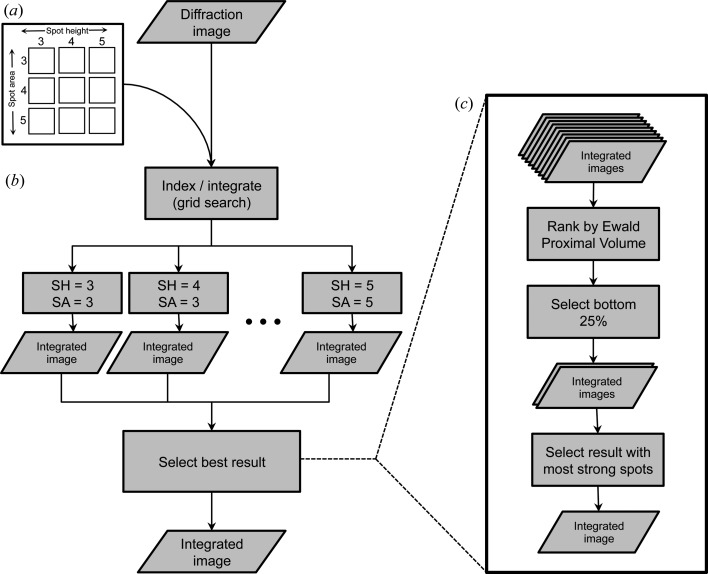
The grid-search and selection process. (*a*) The user supplies the range of spot-finding parameters to be tested. (*b*) *IOTA* carries out spot finding, indexing, lattice model refinement and integration of the diffraction image using every possible pairwise combination of the spot-finding parameters. (*c*) The best result is selected in two steps: (i) the integration results are ranked by the Ewald proximal volume (see Fig. 4) and the bottom 25% are selected, then (ii) the result with the most strong reflections [*I*/σ(*I*) > 5, or a user-supplied threshold] is selected as the best integrated diffraction image.

Correct indexing and successful integration of a diffraction image by *cctbx.xfel* appear to be very sensitive to the initial spot-finding parameters, including the minimum spot area (the number of connected pixels in a peak) and the peak height (the minimum threshold, expressed as the background-subtracted pixel intensity divided by the background standard deviation). These parameters are used by *DISTL* to identify peaks in the diffraction image as candidate Bragg reflections to be used to deduce the crystal lattice (Zhang *et al.*, 2006 ▸). Thus, a spot-finding optimization procedure that accounts for the natural image-to-image variability of spot shapes and sizes is necessary to improve the success of indexing and integration (Fig. 3 ▸). To this end, the user can provide a range for each of the two spot-finding parameters tested by *IOTA* (Fig. 3 ▸ *a*). This range is defined by supplying a median value for each parameter, as well as the range of values above and below that value which are to be tested. The median values for the spot-finding parameters can be determined by utilizing the program *distl.image_viewer*, a part of *DISTL* distributed in the *cctbx.xfel* suite of software. This utility allows the user to test the initial spot-finding parameters using one or several sample diffraction images. *IOTA* then performs a grid search to test all pairwise combinations of these spot-finding parameters for each diffraction image (Fig. 3 ▸ *b*). For each grid point, a full spot-finding, indexing, refinement and integration procedure is carried out and, unless any of the steps fails to complete, the integration result is retained. All integration results are collected for each image and passed on to the selection step.

#### 2.3.2. Optimal integration result selection   

**Figure 4:**
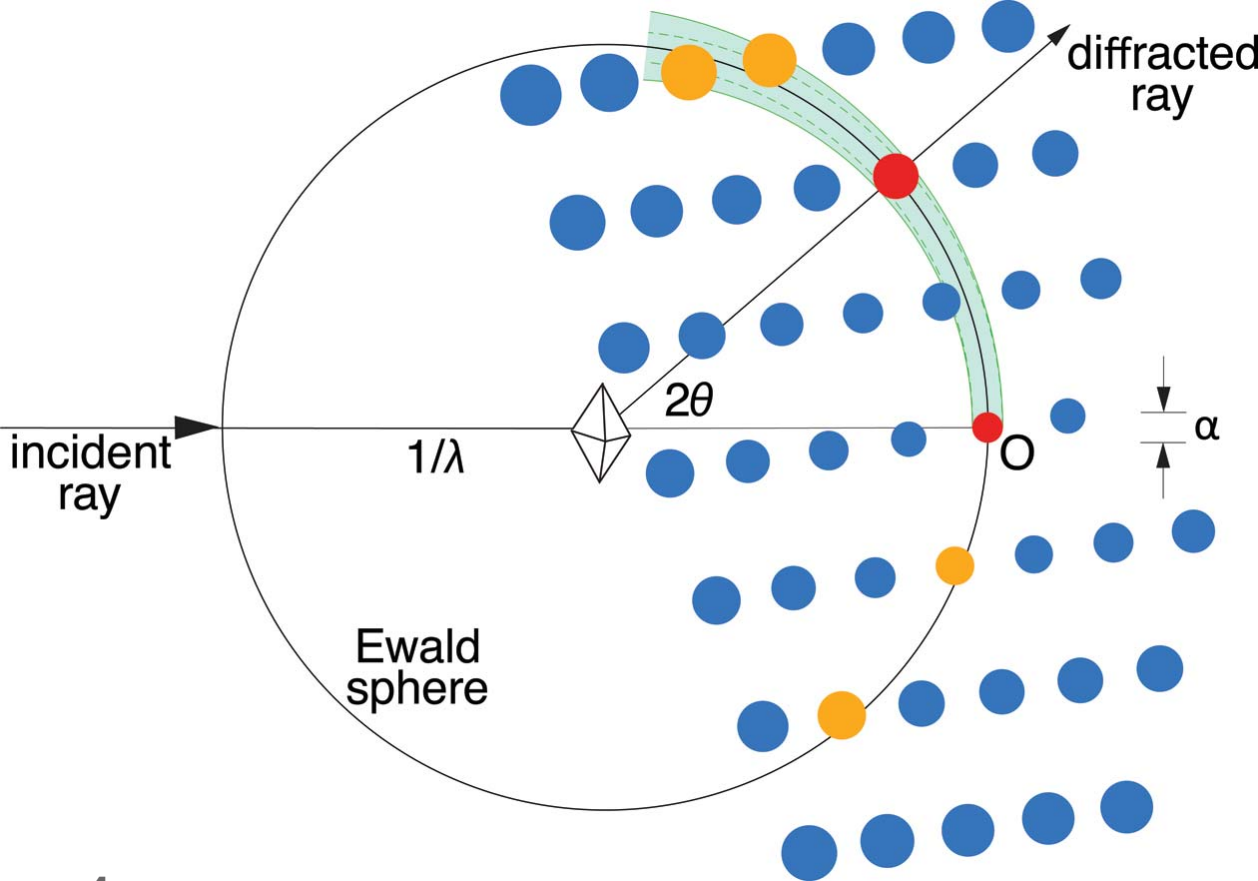
The Ewald proximal volume (EPV) metric is used to evaluate integration success. The EPV (green shaded area) specifies the volume of reciprocal space containing the diffracting centroid positions of recorded reflections. This is equivalent to the assertion that reciprocal lattice points must touch the Ewald sphere to satisfy the diffracting condition. The EPV is defined by rotating the shaded area around the incident beam vector. The diameter α of the reflection *F*_000_ (the red peak at the origin) is determined by the mosaic block size, while the mosaic rotational spread determines how rapidly the spot size increases *versus* distance from the origin. *IOTA* seeks to minimize the overall EPV, while maximizing the number of strong [*I*/σ(*I*) > 5 or user-supplied threshold] integrated reflections within that volume.

To select the best integration result for each diffraction image (Fig. 3 ▸ *c*), it is useful to introduce the concept of an Ewald proximal volume (EPV). The EPV is a property of the crystal’s mosaic structure, as deduced from the set of strong reflections picked by *DISTL*. It is defined here as the volume of reciprocal space that contains Miller index positions whose reciprocal lattice points satisfy the diffraction conditions (Fig. 4 ▸). It is thus a convenient way of visualizing in geometric terms how many Bragg spots *N*_spot_ would be observed by an ideal detector in the absence of background noise,
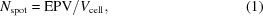
where *V*_cell_ is the primitive-setting volume of the unit cell. EPV and *N*_spot_ are evaluated with a constant user-chosen outer resolution limit *d*_L_ for all spot-integration trials. Within *cctbx.xfel*, the size of the reciprocal lattice points is described by two refinable parameters that arise from the mosaic structure (Nave, 1999 ▸; Sauter *et al.*, 2014 ▸). The effective average size of the coherently scattering mosaic blocks *D* is related to the diameter α of *F*_000_ (the reflection at the origin *O*), with α ≃ 2/*D*, while the effective full-width mosaic rotational angle η determines how rapidly the spot size increases with distance from the origin. Combining these two effects, the full-width reciprocal lattice spot diameter *w* is
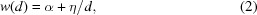
where *d* is the spot resolution appearing in Bragg’s law, λ = 2*d*sinθ, with X-ray wavelength λ and Bragg angle θ. If all other parameters are held constant, the EPV increases when the mosaic rotational angle is increased, or when the mosaic block size is decreased (Nave, 2014 ▸). The volume can be constructed geometrically by rotating the green area in Fig. 4 ▸ by a full 2π circle about the axis defined by the incident beam. Integration may be performed by considering the differential volume element d*V* expressed in spherical coordinates,
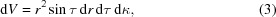
where *r* is the distance from the Ewald sphere center, τ = 2θ is the angle between the incident beam and the diffracted ray, and κ is the rotation angle about the incident beam axis. This is simply the volume element found in elementary textbooks (Lorrain & Corson, 1970 ▸). The integration limits are chosen as follows:
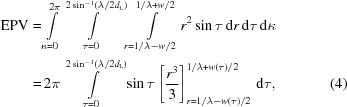
explicitly noting the functional dependence of *w* on τ. The integral is evaluated numerically in the absence of a clear analytical simplification. By sampling a grid of spot-finding parameters, the user chooses differing sets of candidate Bragg spots which, after indexing, produce crystal models with distinct mosaic disorder parameters and thus distinct values for the EPV.

The optimal crystal model is chosen to be the one that best fits the diffraction data, including any weak Bragg reflections that may be below the background standard deviation. Stated differently, the best model has the smallest EPV, provided that the volume is large enough to include the reciprocal lattice points that are actually observed as reflections in the diffraction pattern. Finding the grid-search condition yielding the optimal crystal model is not necessarily trivial, considering the frequent presence of background intensity that exceeds the Bragg signal. However, the following two-step heuristic was found to be useful: (i) the 25% of integration results with the smallest EPV are selected from the complete set (Fig. 3 ▸ *c*, top); (ii) the one integration result with the highest number of strong integrated reflections [*i.e.* with *I*/σ(*I*) above a specified threshold] is selected from the ‘smallest EPV’ fraction (Fig. 3 ▸ *c*, bottom). The final set of integrated intensities, derived from a single diffraction image using the best spot-finding parameters for this image, is then retained and used in the final merged data set (Fig. 3 ▸ *b*).

### 2.4. Unmerged diffraction data analysis   

**Figure 5:**
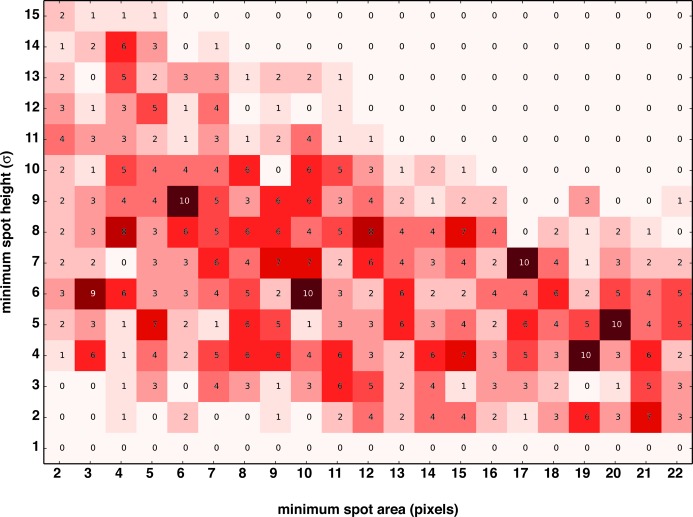
A demonstration of how the optimal spot-finding parameter combinations differ between individual diffraction images. The distribution of the optimal parameter combinations over 705 successfully integrated diffraction images is shown as a heat map. Each diffraction image was indexed and integrated using every possible combination of the two spot-finding parameters; only the selected ‘best’ integration results (one per image) were used to construct the heat map. The color of each square represents the number of optimally integrated diffraction images for this combination of spot-finding parameters, also shown inside the square. Note that no dominant combination of spot-finding parameters effective for the majority of images is found by the grid search, indicating that they should be determined separately for each diffraction image.

Following the integration step, *IOTA* partitions the set of integrated diffraction images into groups (‘clusters’) with similar unit-cell parameters (Fig. 2 ▸ *c*) using the hierarchical clustering toolkit described by Zeldin *et al.* (2015 ▸). All clustering results are output, with the most prevalent result (*i.e.* the unit cell corresponding to the cluster with the most members) marked. This information can then be used to narrow the selection of optimal spot-finding parameters (described in §2.3.1) in subsequent grid-search rounds. Additionally, *IOTA* will provide a summary indicating the range of successful spot-finding parameter combinations, the distribution of limiting resolutions of the individual diffraction images and an overall summary of integration results. *IOTA* also provides several graphical analytics, such as (i) a scatter plot of refined direct-beam coordinates for the diffraction image set, which can assist in detecting mis-indexed frames; (ii) a ‘trumpet’ plot of mosaic disorder in the crystal for each image, as presented in Fig. 7 of Sauter *et al.* (2014 ▸); (iii) an image file with an overlay of the best integration result and the original diffraction image for visualization of the integration results for each image; and (iv) a spot-finding grid-search heat map (Fig. 5 ▸).

### 2.5. Interface with post-refinement software   

*IOTA* automatically populates a parameter file for the program *PRIME* (Fig. 2 ▸ *c*), which post-refines crystal orientation, unit-cell parameters and mosaicity parameters in order to model accurately the reflection partiality, corrects the partial observations to their full equivalents, and scales and merges the data (Uervirojnangkoorn *et al.*, 2015 ▸). In cases where a clear dominant cluster is present, as determined by the Andrews–Bernstein algorithm (Andrews & Bernstein, 2014 ▸) implemented in the unit-cell clustering software (Zeldin *et al.*, 2015 ▸), the list of integrated diffraction images composing this cluster is used as input to *PRIME*. When more than one Laue class is possible given the Bravais lattice (*e.g.* *P*4/*m* and *P*4/*mmm* for a primitive tetragonal lattice), *IOTA* will select the lowest-symmetry Laue class associated with the Bravais lattice, which is then used for scaling, merging and post-refinement in *PRIME*. The user has an option of manually editing the *PRIME* input file with any additional information (such as crystal symmetry and/or unit-cell dimensions different from those output by *IOTA*) that is known *a priori.* This allows the user to perform both raw diffraction-image processing and data merging, scaling and post-refinement in two easy steps with minimal human involvement. Furthermore, information from *PRIME* can be used to adjust the indexing/integration parameters of *cctbx.xfel* and *IOTA*, enabling the user to explore multiple diffraction data-processing strategies in a controlled and coordinated manner.

### 2.6. Examples   

Initial testing of the impact of spot-finding parameters on indexing and integration was performed using serial diffraction images from HEWL microcrystals (≤15 µm). The images were obtained at room temperature using a synchrotron beamline with zero crystal rotation, with crystals delivered to the beam by means of a micro-patterned silicon nitride device (Murray *et al.*, 2015 ▸). Diffraction data were recorded using a Dectris PILATUS 6M detector (Fig. 1 ▸).

For a test case involving a previously uncharacterized crystal system, we made use of the XFEL-derived diffraction images from crystals of the synaptotagmin-1 (Syt1)/SNARE complex. The results of this test eventually led to the structure determination and refinement of the Syt1/SNARE crystal structure from XFEL diffraction data (Zhou *et al.*, 2015 ▸). The Syt1/SNARE crystals were immobilized in conventional cryo-loops at 100 K. Diffraction data were obtained using a fixed-target goniometer setup (Cohen *et al.*, 2014 ▸) and a Rayonix MX325-HE detector on the X-ray Pump Probe (XPP) endstation of the LCLS.

## 3. Results and discussion   

The grid-search approach performed well with a small set of XFEL-derived diffraction images obtained from thin plate-like crystals of the Syt1/SNARE complex (Zhou *et al.*, 2015 ▸). A total of 789 XFEL exposures were obtained from 148 crystals, of which 54 contained no useful diffraction. Complicating the analysis of the diffraction data, the Syt1/SNARE complex crystallizes in two distinct but very similar crystal forms, both in primitive orthorhombic space groups (*P*2_1_2_1_2 and *P*2_1_2_1_2_1_) with very similar unit-cell dimensions (*a* = 69.1, *b* = 171.6 and *c* = 146.9 Å, and *a* = 69.6, *b* = 171.1 and *c* = 291.9 Å, respectively). The major difference between these unit-cell types is the approximate doubling of the *c* axis; consequently, we termed these crystal forms ‘short unit cell’ and ‘long unit cell’, respectively. It was possible to separate the two crystal forms using a clustering method (Zeldin *et al.*, 2015 ▸). Table 1 ▸ summarizes the results of indexing and integration with and without spot-finding parameter optimization, using identical *cctbx.xfel* settings. In addition, each experiment was repeated while applying a Bravais lattice filter (primitive orthorhombic), using unit-cell information determined from the first round of indexing and integration.

As outlined above, initial spot-finding parameters were chosen using the program *distl.image_viewer*. However, this process is time consuming even for a single diffraction image and would be prohibitive for a data set containing hundreds or thousands of images, so we performed these initial tests only for a small random subset of the images. As a result of these tests, we chose a minimal spot-height parameter of 8 ± 7σ (in units of the background noise standard deviation) and a minimal spot area of 12 ± 10 pixels as the starting median parameters; this approach yielded a 15 × 21 grid that was used for the spot-finding parameter grid search. A heat map of the spot-finding parameter distribution illustrates the high image-to-image variability of the combinations that lead to successful integration (Fig. 5 ▸). Notably, while several ‘hot spots’ emerge, suggesting that there are parameter combinations that are optimal for a substantial subset of diffraction images, there are never more than ten integration successes for a given combination. This result suggests that a single optimal combination applicable to the entire set of diffraction images cannot be obtained using *cctbx.xfel*, and thus must instead be individually determined for each individual diffraction image.

**Figure 6:**
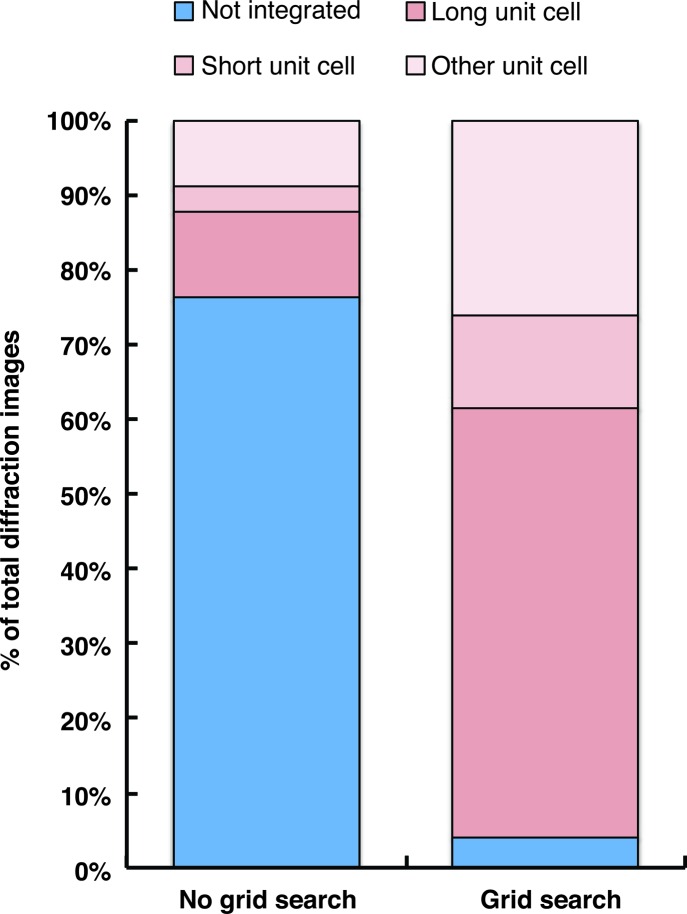
Spot-finding parameter optimization improves the success of diffraction-image integration for the XFEL diffraction data set of the Syt1/SNARE complex. Integration results with and without a spot-finding parameter grid search (without the Bravais lattice filter applied) are plotted as a percentage of the total collected diffraction images. A spot area of 10 pixels and spot height of 8σ were used for the integrations without grid search. A spot area range of 2–22 pixels and spot height range of 1–15σ were used for the grid searches.

Without spot-finding parameter optimization, ∼25% (195) of the diffraction images could be integrated (Fig. 6 ▸, Table 1 ▸). Only ∼10% (82) of the diffraction images were indexed with the correct unit-cell dimensions for the ‘long unit cell’ type (Table 1 ▸, columns 2 and 3). In contrast, spot-finding parameter optimization substantially increased the percentage of total integrated diffraction images to ∼89% (705), with ∼54% (423) of the images indexed in the long unit cell. Here, the number of long unit cell images actually increased after the Bravais lattice filter was applied, as incorrect solutions were discarded in several of the cases, thus biasing integration result selection towards the correct solution (Table 1 ▸, columns 4 and 5). In summary, these results demonstrate the utility of the grid-search procedure, even with the most recent version of *cctbx.xfel*, which features a number of notable improvements to the indexing and integration modules (Lyubimov *et al.*, in preparation).

The completeness of the merged diffraction data set improved from 60.2% (1.8 observations per Miller index) without spot-finding parameter optimization to 97.7% (6.3 observations per Miller index) with spot-finding parameter optimization (Table 1 ▸). It should be noted that we had previously performed the grid-search procedure on the same set of diffraction images using older versions of *IOTA* and *cctbx.xfel* and obtained similar improvements *versus* processing without spot-finding parameter optimization. The resulting merged diffraction data set was used to detemine a 3.5 Å XFEL structure of the Syt1/SNARE complex, which has been discussed in detail elsewhere (Zhou *et al.*, 2015 ▸). Subsequent rounds of fine-tuning the indexing, integration, long unit cell image selection, scaling and post-refinement settings further improved the final merged data set derived from 328 diffraction images, resulting in a completeness of 97.1% (5.6 observations per Miller index) to a limiting resolution of 3.5 Å. These further improvements are specific to the *cctbx.xfel* and *PRIME* packages, and will be discussed elsewhere (Lyubimov *et al.*, in preparation).

## 4. Conclusions   

The high image-to-image variability inherent in a typical SFX data set makes reliable indexing and integration of each individual diffraction image difficult, with many diffraction images either mis-indexed and integrated poorly, or not indexed or integrated at all. Here we have presented a grid-search-based optimization of spot-finding parameters for each individual diffraction image and shown that it substantially increases the overall success rate of indexing and integration, and improves the quality of the merged and post-refined diffraction data set. The integration optimization module, in conjunction with image pre-processing and analysis modules, has been implemented in the program *IOTA*, which is distributed as part of the *cctbx.xfel* package (http://cci.lbl.gov/xfel) under an open-source license.

## Figures and Tables

**Table 1:** The impact of spot-finding parameter optimization for the XFEL diffraction data set of the Syt1/SNARE complex

	No grid search, no filter†	No grid search, Bravais lattice filter‡	Grid search, no filter†	Grid search, Bravais lattice filter‡
Total images	789	789	789	789
No diffraction	54	54	54	54
Not integrated	540	540	30	30
Failed filter§	N/A	83	N/A	56
Long unit cell (222)	82	70	423	482
Short unit cell (222)	24	20	90	104
Other unit cells and/or Bravais lattices¶	89	22	192	64
Completeness for long unit-cell crystal form (%)††	60.2	59.9	97.7	98.4
Mean No. of observations for long unit-cell crystal form††	1.8	1.8	6.3	8.0

† All entries were retained from the full set of successful integration results for each image.

‡ Only entries that yielded a primitive orthorhombic Bravais lattice were retained from a set of successful integration results for each diffraction image.

§ This fraction contains diffraction images for which none of the integration results yielded the primitive orthorhombic Bravais lattice.

¶ Images indexed with parameters that fall within neither long nor short unit-cell clusters.

†† Reported to a limiting resolution of 3.5 Å. Further optimization of integration and merging/post-refinement parameters yielded the merged data set used to solve and refine the XFEL-based Syt1/SNARE structure (Zhou *et al.*, 2015 ▸).
